# Crosstalk of Autophagy and Apoptosis

**DOI:** 10.3390/cells11091479

**Published:** 2022-04-28

**Authors:** Maurizio Sorice

**Affiliations:** Department of Experimental Medicine, “Sapienza” University of Rome, 00161 Rome, Italy; maurizio.sorice@uniroma1.it; Tel.: +39-6-49972675

**Keywords:** autophagy, apoptosis, cell signaling, lipid rafts, calcium, PINK1, cancer therapy

## Abstract

Autophagy and apoptosis represent two fundamental pathophysiological mechanisms of cell fate regulation. However, the signaling pathways of these processes are significantly interconnected through various mechanisms of crosstalk. Indeed, autophagy/apoptosis crosstalk is still an emerging field, in which an increasing number of molecules are involved, including, for example, PINK1 and ERLINs. On the other hand, this crosstalk involves signal transduction pathways which are strongly dependent on Ca^2+^. Interestingly, crosstalk between autophagy and apoptosis impacts several pathologies, including multiple rheumatic diseases. The purpose of this Special Issue is also to investigate the bioactive properties of drugs with antitumor activity, focusing particularly on the role of anthraquinone derivatives in the regulation of cell death and autophagy crosstalk. This Special Issue of *Cells* brings together the most recent advances in understanding the various aspects of crosstalk between autophagy and apoptosis and the interconnected signaling pathways, implying therapeutic perspectives for the utility of its modulation in an anti-cancer setting.

Autophagy and apoptosis represent two fundamental pathophysiological mechanisms of cell fate regulation. Novel evidence supports the view of a strict interconnection of the signaling pathways of these processes through various mechanisms of crosstalk that several studies contributed to elucidating. An example is provided by the study of Brunelli et al. who investigated the role of PINK1 in the crosstalk between autophagy and apoptosis [1]. They observed that the interaction between PINK1 and Beclin1 is responsible for initiating autophagosome formation, demonstrating an anti-apoptotic function of PINK1 in SH-SY5Y neuroblastoma cells. In this way, they assayed the pro-survival activity of PINK1 in response to staurosporine, which is able to promote apoptosis but not mitophagy. Notably, the authors highlight a new activity for PINK1 in the impairment of pro-apoptotic cleavage of Beclin1 upon staurosporine treatment. Interestingly, a phase of increased autophagy precedes staurosporine-induced apoptosis. Concerning this, PINK1 regulates the switch from autophagy. Indeed, PINK1 levels progressively decrease following treatment, inducing this switch. The PINK1–Beclin1 interaction may be a key role in this process since mutants that are unable to interact do not show any anti-apoptotic effect.

On the other hand, the crosstalk of autophagy and apoptosis involves signal transduction pathways which are strongly dependent on Ca^2+^ [2]. Indeed, Ca^2+^ functions as a second messenger are critical in coordinating fundamental physiological functions, including cell survival and growth, neuronal development and/or the maintenance of cell functions. The coordination among proteins/pumps/Ca^2+^ channels and Ca^2+^ storage in different organelles is critical in preserving cytosolic Ca^2+^ levels that keep the spatial resolution needed for cellular homeostasis. Ca^2+^ homeostasis is regulated by a store-operated Ca^2+^ entry (SOCE) mechanism that is activated by the depletion of Ca^2+^ from internal ER stores. Ca^2+^ has been shown to control opposing functions, such as autophagy, that promote cell survival; on the other hand, Ca^2+^ also regulates programmed cell death processes, including apoptosis. Recently, it has been evident that a complex network of lipid–lipid and lipid–protein interactions promotes the activation of a variety of signaling pathways regulating cell homeostasis [3]. In this way, specific plasma membrane microdomains, named lipid rafts, regulate a variety of signal transduction pathways involved in specific cellular programs, including proliferation, apoptosis, differentiation, stress responses and autophagy, thus determining cell fate [4]. However, lipid rafts are not only in the plasma membrane [5], but are also present in the membrane of intracellular organelles, including ER, Golgi apparatus, endosomes, lysosomes. At these sites, key reactions can be catalyzed with a significant impact on the regulation of intracellular trafficking and sorting, cholesterol homeostasis and cell fate. Mitochondria-associated membranes (MAMs) have been classified as critical “hubs” in the regulation of apoptosis, autophagy and tumor growth. Recently, the discovery of lipid rafts as physical and functional platforms within MAMs, contributed to the elucidation of mechanisms underlying the early steps of the autophagic process [6]. In particular, it is emerging that ER lipid raft-like microdomain proteins, i.e., ER lipid raft-associated protein 1 (ERLIN1) and 2 (ERLIN2), may drive mitochondria-ER crosstalk under both physiological and pathological conditions by association with MAMs, thus regulating survival and death. Manganelli et al. describe the role of ERLINs in determining cell fate by controlling the “interchange” between apoptosis and autophagy pathways [7]. The proposed role of ERLINs in the degradation of the calcium channel (inositol 1,4,5-triphosphate receptor), could explain their role in the autophagy mechanism, adding a significant impact on the pathogenesis of several human diseases.

Indeed, crosstalk between autophagy and apoptosis impacts several pathologies, including multiple rheumatic diseases. Since mitochondria are significant regulators in maintaining cartilage homeostasis, turnover of mitochondria through mitochondrial biogenesis and mitochondrial degradation may play an important role in the pathogenesis of osteoarthritis (OA). Kim et al. discuss the role of mitochondrial dysfunction in OA pathogenesis, identifying the peroxisome proliferator-activated receptor-gamma co-activator 1-alpha (PGC1α) as a potent regulator [8]. They demonstrated that the loss of PGC1α in chondrocytes due to upregulation of miR-126-5p during OA pathogenesis culminated in the activation of PRKN-independent mitophagy through BNIP3 upregulation and stimulated cartilage degradation and apoptotic death of chondrocytes. In addition, the dysregulated balance between autophagy and apoptosis may be involved in the pathogenesis of rheumatoid arthritis, systemic lupus erythematosus and Sjogren’s syndrome [9]. Indeed, it may regulate the survival of immune cells, peptide citrullination, the presentation of autoantigens, and B- and T-cell maturation. Notably, some currently used disease-modifying antirheumatic drugs (DMARDs), including glucocorticoids, hydroxychloroquine, rapamycin, anti-TNFα and Jak inhibitors may act through autophagy/apoptosis pathways.

The purpose of this Special Issue is also to investigate the bioactive properties of drugs with antitumor activities, focusing on their role in the regulation of autophagy and cell death crosstalk that triggers the uncontrolled expansion of tumor cells. In this vein, an example is represented by anthraquinone derivatives, which may affect autophagy and/or apoptosis and promote oxidative changes in cancer cells [10,11]. In particular, Trybus et al. [10] showed that physcion induces an increased number of lysosomes, autophagy vacuoles, and upregulation of the LC3 protein level. At the same time, the pro-apoptotic effect of physcion was identified and this mechanism was dependent on caspases 3/7 activation together with a reduction in Bcl-2 expression. The induction of apoptosis and autophagy may represent a common mechanism for the anti-tumor effect of physcion. Morgan et al. analyzed the effects of a wider panel of anthraquinone derivatives tested in the colon, prostate, liver and cervical cancer cell lines [11]. Among these compounds, 1,4-bis(benzyloxy)-2,3-bis(hydroxymethyl)anthracene-9,10-dione was shown to induce apoptosis through the activation of caspases and the production of PARP. Moreover, autophagy also plays a role in its mechanism of action since it activates the conversion of LC3A/B-I to LC3A/B-II and causes p70 s6 kinase phosphorylation.

Altogether, the articles in this Special Issue further improve the knowledge of the mechanisms regulating cell fate, analyzing either the signaling pathways regulating cell death and survival or the bioactive properties of drugs acting on the crosstalk between autophagy and apoptosis. This understanding may drive the development of a new therapeutic strategy for cancer and/or autoimmune diseases.
